# Book Reviews

**Published:** 1821-07

**Authors:** 


### CRITICAL ANALYSES

OF

### RECENT PUBLICATIONS, IN THE DIFFERENT BRANCHES OF MEDICINE AND SURGERY.

"I would have men know, that, though I reprehend the easie passing over of the causes of things "by ascribing them to secret and hidden vertues and properties; (for this hath arrested and laid "asleepe all true enquiry and indications;) yet I doe not understand but that, in the practical "part of knowledge, much will be left to experience and probation, whereunto indication cannot "so fully reach: and this not only in *specie*, but in *individuo*. Yet it was well said, *Vere scire* "*esse per causas scire*."—BACON.

*A Treatise on Indigestion, and its Consequences, called Nervous and Bilious Complaints; with Observations on the Organic Diseases in which they sometimes terminate.* By A. P. W. PHILIP, M.D. F.R.S.ED. &c. 8vo. pp. 363. T. and G. Underwood. London, 1821.

THE author, in a Preface, after having stated the objects of this work, says that, in the composition of it, "recourse has not been had to the works of others. It cannot, therefore, be regarded as an attempt to present to the reader the sum of our knowledge on the subject. I offer it simply as the result of my observations, and the reflections suggested by them, during a space of twenty-five years." It is because the statement in the last sentence is so strictly correct, that the perusal of this book must prove particularly interesting and gratifying to the well-informed part of the profession. Medical writers are too apt to imagine that the best way of presenting to the public what they suppose to be their own original observations and opinions on any subject, is to develop them in a formal general treatise, professing to give a summary of the most important of the extant knowledge; by which conduct they also hope to perpetuate their claims to merit in a *classic* work: it is only necessary to inspect the libraries of men of good sense and education, and to consider what are the works which have gained their authors permanent and estimable reputation, in order to perceive how erroneous are those notions. Indeed, we think it is obvious that the most favourable way by which a man may fail to attain the credit he deserves for his contributions to science, is to diffuse them in a compilation of the sort just alluded to: they will here be likely to escape the attention of men of due attainments, who will but rarely seek what is proper to an author, when it is so dispersed amongst what they have already become acquainted with, and who, also, have the judgment to know that the best, if not the only, way of acquiring proper information of matters relating to subjects so abstruse as are those of medicine, is that of perusing the relations of the actual discoverers of original knowledge; and it is the decisions of men thus qualified that, sooner or later, lead the voice of the public in the expression of praise as well as blame. When knowledge on any subject has been gradually and slowly accumulating for many ages, and is still only widely dispersed in the writings of men of various nations, a compilation presenting a satisfactory account of it must become of great utility, and the construction of such a work, professing only to be what it really is, is so meritorious an effort, that hardly any man should think it unworthy of his powers: it was with such works that Priestley began his scientific career: but it is a pitiful spirit of ambition that leads a man to proffer what he knows to be plagiarisms, as his own acquirements; and, which is still worse, to try to make the weaker part of the public, and the more shallow-headed and indolent portion of the profession, receive the results of his superficial views as a profound and sufficient exposition of what is really known. This, however, is what has, of late especially, been so often seen in books on indigestion and its consequences; whilst, notwithstanding the immense number of such writings, these morbid states are as little understood as almost any disorder of the human body.

Dr. Philip's treatise is really his own; his observations, as well as his inferences from them, are, to a great extent, peculiar to himself; and those which the reader may recognize as similar to some which he may have acquired or met with in former writers, are still marked by a degree of precision and unison with the rest, that characterize them as the author's original attainments. We have already hinted that we think this treatise a production of very considerable value; but we shall now, by passing the several parts of it in review, endeavour to point out more particularly in what we consider its merits chiefly to consist; or, in other words, to show how much it contributes to our knowledge of the history and means of cure of indigestion.

The author has arranged the various cases of which he treats under the denomination of *indigestion*, "because, (he says,) the symptoms of this derangement form a more or less prominent feature in all, and all begin with these symptoms."—"I prefer, (he continues), this term to dyspepsia, which has been employed by medical writers to express a disease much less varied, and of much less extent, than that I am about to treat of. Indigestion, therefore, in the sense in which I shall use this term, is not synonymous with dyspepsia, but includes it."

"By a disease we mean, (Dr. Philip adds,) not only that collection of symptoms which are present at any one time, but also those which appear in succession, arising from the same source. We shall find indigestion the most varied of all diseases. Beginning from simple and apparently unimportant deviations from health, it gradually becomes so complicated, and often, at length, so undermines every power of the system, that it is difficult to give a view of its symptoms, which shall be at once sufficiently full and distinct. It is an affection of the central part of a most complicated structure, capable of influencing even its remotest parts, and each through many channels and in various ways."

According to this explanation of the sense in which the author uses the term indigestion, there can be no valid objection to his application of it: that which originates from the real meaning of this word and of dyspepsia, is thus obviated; but the want of a more proper appellation for this morbid state than that which expresses only one consequence of it when it exists in its extreme degree, is, at the same time, rendered strikingly apparent. It is sufficient, however, for our present purpose to have shown in what sense the author employs the term he has adopted.

The treatise, properly, commences with an account of the *symptoms of indigestion:* the first order of which the author divides into such as immediately arise from the undigested food itself; and such as depend on the state of the stomach and bowels which causes the disease, and the irritation of their nerves, occasioned by the undigested food or their own vitiated secretions: of these, one of the most remarkable is that state of general debility which is so constant an attendant of indigestion, and the origin of which the author illustrates by noticing the effects of an emetic; and "when we consider," he adds, "that the causes which disorder the powers of the stomach in indigestion are of a more permanent nature, and that the contents which irritate its surface, although often removed, as in the case of the emetic, are soon re-produced, we shall find little difficulty in perceiving the general rationale of the symptoms of this disease." Sometimes, however, the symptoms which arise immediately from undigested food,—as flatulence, distention of the stomach and bowels, and acid, oily, and putrescent eructations,—will exist, at the commencement of the disease, for a considerable period, without being attended by any sensible general disorder; "from which it appears," the author remarks, "that the functions of the stomach may, for a certain time, be so disordered as to produce a feeble or otherwise vitiated secretion, without in any other way very sensibly affecting the functions of the system;" and hence the affection proceeds, with perhaps some interruptions from the efficacy of aperient medicines, and the like, until the patient feels some depression of strength, before his attention is seriously directed to its existence. This lapse of the first stage of the disease before we are consulted would appear, according to the author's view of the general origin and progress, and the modifications it suffers in its course, to be worthy of particular consideration. With this state of general debility there is, very frequently, more or less despondency or great anxiety of mind. The next important step is announced by an alteration in the appearance of the alvine discharge, and which we have reason to believe arises chiefly from a morbid state of the bile, and, probably, too, of the secretions from the intestines themselves. Then are added, what are called the nervous and bilious symptoms of the malady. After having considered these states in a more particular manner, the author notices the properties of the urine. Some of his experiments have led him to infer, that when acid greatly prevails in the stomach and bowels, or the skin becomes more inactive than usual, so that it does not freely throw off the acid, which, he thinks it appears from his experiments, always passes by this organ, a red deposition, which consists of lithic acid, takes place from the urine after it has stood for some time: and that, when the skin has been unusually excited, or an alkalescent state of the stomach and bowels prevails, the urine becomes turbid, and deposits a white sediment, which has been ascertained, by Dr. Wollaston, to consist of the phosphates.

The state of urine abounding with lithic acid, is that, the author says, most apt to appear in indigestion; and this fluid is, also, sometimes "covered with a very thin oily film, which appears to arise from an imperfect state of the assimilating process." The author mentions several curious circumstances connected with the vicarious functions of the skin, with the intestines as well as with the kidneys; showing what copious evacuations will take place from the bowels, though not much is taken as food, when the skin is dry and shrivelled: and, on the other hand, he notices the fact, that, in the case of an individual who ate daily twelve or fifteen pounds of raw meat, the alvine discharge was but little, if at all, greater than usual; yet the subject continued thin, the superfluous quantity of food appearing to be carried off by profuse night-sweats.

We cannot follow the author through his account of the more severe symptoms of disorder of the stomach and bowels that frequently accompany this stage of indigestion; neither can we select from it any particular observations strikingly novel. We can only remark that it is sufficiently expressive, except that no mention is made of *incubus* as a symptom of indigestion. That incubus is a symptom of the latter affection, seems to be proved by the fact that, in many persons subject to indigestion, it occurs only after they have eaten supper. We shall also remark, that the author very properly insists on the importance of distinguishing the debility which results from the want of due nutrition from that which accompanies the commencement of the disease, and arises merely from the morbid condition of the stomach, or from painful impressions on this organ.

The thoracic symptoms in this stage of the disease consist of more or less permanent dyspnœa; a dry and harassing cough; pain in the side, most frequently the left, and which is sometimes seated in the muscles of the chest; and, in some few cases, palpitation of the heart. The symptoms alluded to in the foregoing remarks appertain to the first stage of the disease, and which, as we shall hereafter show in a more particular manner, the author considers chiefly to consist and originate from a state of debility of the stomach; but, he says,

"At various periods of the disease, for the most part after repeated derangement of the hepatic function, comes on a permanent tenderness on pressure, sometimes but slight, of the soft parts close to the edge of the cartilages of the false ribs on the right side, after they have turned upwards to be joined to the sternum. This spot is often very circumscribed, and always lies about half-way between the end of the sternum and the place at which the lowest of the cartilages begins to ascend; and the cartilage itself near the tender part often becomes very tender, not unfrequently, indeed, much more so than the soft parts. The patient, in general, is not aware of this tenderness till it is pointed out by the physician.

"This symptom never exists long, and to any considerable degree, without the pulse becoming hard, and it often, at the same time, becomes rather more frequent than in health. There is no other symptom of the disease before us to which I am so anxious to call the reader's attention, as to what I have here termed a hard pulse, because on it much of the proper treatment seems to depend. It sometimes happens, especially when the tenderness in the epigastrium is considerable, that the pulse becomes such as would on all occasions obtain the name of hard; but more frequently the hardness is only to be distinctly perceived by examining the pulse in a particular way.

"Those who have been much in the habit of examining the different states of the pulse, must be aware that its hardness is most perceptible when a slight degree of pressure is employed. A certain degree, by greatly compressing the vessel, will give some feeling of softness to the hardest pulse; and a slight degree of hardness is not perceptible with the pressure generally employed in feeling the pulse. If the pressure be gradually lessened till it comes to nothing, it often happens that a distinct hardness of pulse will be felt before the pulse wholly vanishes under the finger, when no hardness can be distinguished in the usual way of feeling it.

"This is in no degree the case in a healthy pulse, nor even in the first stage of the disease we are considering. But when the tenderness of the epigastrium is at all a prominent feature, it may always be perceived: that is, there is then a certain degree of pressure, sometimes very slight, under which the pulse gives a decidedly wiry sensation to the finger, the degree of pressure under which the hardness may be perceived denoting its degree.

"I consider the occurrence of the tenderness of the epigastrium and hard pulse as dividing the disease into two stages; because, from the time of their appearance, at whatever period this happens, we shall find its nature, and consequently the plan of treatment suited to it, changed.

"These symptoms are generally accompanied with others, indicating some degree of feverishness. The chilliness of which the patient has long complained is now sometimes, and independently of any change of temperature in the surrounding medium, interrupted by languid and oppressive fits of heat; and the hands and feet, instead of being uniformly cold, as in the earlier stages, often burn, particularly during the first part of the night, while at other times they are more obstinately cold. The thirst also often increases; and sometimes there is a tendency to partial sweats in the morning, especially if the patient lie longer than usual."

The tenderness of the epigastrium, having existed for some time, generally becomes at length attended by some degree of fullness in the part, and extends downwards along the edge of the cartilages of the ribs. This fullness of the right hypochondrium is permanent, and thus differs from that which occurs in earlier periods, and which is generally relieved, and sometimes removed, by the effects of cathartics; and not unfrequently spontaneously disappears, and returns again.

After indigestion has proceeded to this extent, it is not unfrequent for the disease of some organ, or part which had previously participated in the effects of the disorder of the stomach, now to become more obstinate and severe, and at length the most prominent affection; and, indeed, the chief and most serious malady, and such as cannot be removed without an appropriate mode of treatment directed expressly to the part itself.

The symptoms enumerated as those of the second stage mark conditions which cannot, the author observes, continue long without change of structure, which ushers in what he considers as the third stage of the disease; and, he remarks, "it is a curious fact, and one of the greatest importance in the treatment, that the organic affection rarely takes place in the original seat of the disease, but in other organs with which the stomach sympathizes,—the liver, pancreas, spleen, mesenteric glands, lower bowels, heart, lungs, brain, &c.;" and, when the body is examined after death, the patient is often said to have died of disease of some of these parts. Another very interesting remark of the author is, that, when disease fixes decidedly on one organ, the others are, to a certain degree, and sometimes wholly, relieved. The establishment of the secondary affection generally relieves the dyspeptic symptoms; and even a secondary disease may be removed by another supervening on it. What is merely nervous irritation in organs affected by sympathy, will at length terminate in inflammation; and the recognizance of such a change having taken place is of great importance in regard to the treatment of the disease. These, and various other more or less interesting circumstances in the history of the disease, are noticed by the author; and it is from his having, justly, attributed a high degree of importance to them, that he has been induced to make his observations on the treatment, in which they are particularly considered, occupy the greater part of the work. It is, indeed, we think, the manner in which he appreciates these conditions in a therapeutical point of view, that constitutes the chief practical value, as well as the more original part, of his treatise.

With regard to the circumstances which dispose the sympathetic disease to affect one part in preference to another, the author says, "we have reason to believe that this is chiefly determined by different parts in different individuals being more liable to disease than others, and therefore feeling more the cause of irritation which affects the whole system. Thus, in children, who are disposed to inflammation and subsequent effusion in the ventricles of the brain, indigestion often terminates in hydrencephalus internus. From about fifteen to thirty-five years of age, the disposition to affections of the lungs is greatest, and it often produces phthisis. At a more advanced period, a tendency to disease of the rectum prevails, and in old age to affections of the heart and head,—the latter, however, of a different nature from those to which children are subject; and we still observe the tendency of indigestion to produce the disease to which the system is disposed, whatever be its seat."

When noticing more particularly those particular secondary affections, the author gives a hint of his notions of the pathology of gout. "When a tendency to this disease exists," he says, "it may be induced by any cause that produces, and for a certain time keeps up, indigestion. In some the disposition to gout is so great, that it appears without being preceded by symptoms of derangement in the first passages; but, in the majority of cases, it is preceded by these symptoms, and the tendency to them seems to constitute a considerable part of the hereditary disposition to gout."

This pathology nearly agrees with that of Broussais, who regards gout as merely a sympathetic affection of disease of the digestive organs, as we have already mentioned in this Journal. It is on the principle of the secondary disease relieving the primary, or other secondary diseases, in indigestion, that depends the danger which attends an interruption of the regular fits of gout. "The sympathetic disease," Dr. Philip says,

"being prevented from taking the course which the disposition to affection of the extremities give it, seizes on the part, generally an internal one, which next to these is most liable to disease; and, on the other hand, if any thing so affects any of the vital parts during a fit of the gout, as to render it considerably the weakest part, the sympathetic disease sometimes leaves the joints, and seizes on the internal part, producing what is called retrocedent gout. It is evident that the risk of both these accidents will be greatest where the powers of the system are most impaired." We have a voluminous modern work on Gout, known to most medical men, which does not contain so much real information of this disease, nor even so much information of practical utility,—notwithstanding its pretensions,—as those few remarks of Dr. Philip. But, from writers who are not physiologists, we should expect nothing but vague, perplexed, and obscure, delineations of all such diseases as involve any of the more abstruse operations of the animal economy.

The *alternation* of gout and gravel has been a perplexing circumstance to most writers on these diseases. It has already been stated that the formation of lithic acid, to a certain extent, is a frequent symptom of indigestion; and it is sufficiently proved that the ordinary gravel is concreted lithic acid. There is, then, no difficulty in comprehending, after what has been said of the etiology of gout, how this alternation should occur; as it is in the intervals of gout that indigestion chiefly prevails, the affection of the joints relieving that of the stomach.

Having discussed this part of his subject, the author treats of the *causes* of indigestion; and, he remarks, as it is impossible to understand the operation of the remote causes, or the nature of the symptoms of this affection, without the knowledge of the digestive process, our attention must be directed to this subject.

"It has been ascertained, (says the author,) by the experiments of Spallanzani and others, that the stomach secretes a fluid capable, even out of the body, of converting the food into such a mass as that into which it is changed in the stomach, immediately before it is sent into the duodenum. This fact leaves no room to doubt that it is by the agency of the above fluid that the food undergoes the change which is effected on it in the stomach; and it appears, from the observations of Mr. Hunter, that such is the power of this fluid that it often corrodes the stomach itself when deprived of the vital principle by which it is enabled to resist its action. When to these facts we add, that, by means of the muscular power of the stomach, the food, when duly prepared by the action of the gastric fluid, is propelled into the duodenum, we state the sum of our knowledge on this subject. These facts, however, are far from affording an explanation of the digestive process. It does not appear, from the observations of any of the authors here referred to, by what means the fluid which is secreted by the coats of the stomach is uniformly applied to the food, nor upon what principle the food which is prepared for the duodenum is separated from the rest.

"Mr. Hunter found that, even when part of the stomach itself had been dissolved by the gastric fluid, the food last taken remained wholly unchanged. This fact alone is sufficient to militate against the idea assumed by some, that, by the muscular power of the stomach, the food is so moved as to be continually in the act of being mixed together and with the gastric fluid, by which its uniform solution is effected. According to this view of the subject, also, it would be difficult to account for the gradual discharge of the contents of the stomach into the duodenum. This intestine must either for a long time cease to receive any food from the stomach after every addition made to the contents of the latter, or receive food in every different stage of solution,—an imperfection in a natural process which is opposed by every thing we know of the animal economy."

The results of the author's researches on this subject constitute, as we think, the most valuable of his contributions to physiology. Dr. Philip has examined the stomachs of about a hundred and thirty rabbits immediately after the animals had been killed in the usual way, which is by a blow on the back part of the head, at various periods of digestion. The first thing that strikes the eye on examining the stomachs of rabbits which have lately eaten, the author observes, is that the new is never mixed with the old food. The former is always found in the centre surrounded by the old food, except that, on the upper part between the new food and the smaller curvature of the stomach, there is sometimes little or no old food. The line of separation between the two is distinctly evident. The food which touches the surface of the stomach is more digested than any other found in the same part of the stomach; and (unless the animal has not eaten for a great length of time,*) the food in contact with the surface of the stomach is in very different states of digestion in different parts of the organ. It is least digested in the small curvature, more in the large end, and still more in the middle of the great curvature. The foregoing remarks apply to the cardiac portion of the stomach; the food in the pyloric portion is always found in a state very different from that just described. It is more equally digested; the central parts differing less from those which lie near the surface of the stomach. It is evident, however, that all the change effected in the stomach is not completed when the food enters this portion of it, because it is found the more digested the nearer it approaches to the pylorus. It appears that, in proportion as the food is digested, it is moved along the great curvature, where the change in it is rendered more perfect, to the pyloric

* It appears that the stomach of the rabbit is not able so to contract as to obliterate its cavity, for some part of its contents always remains.

portion. Thus, the layer of food lying next the surface of the stomach is first digested; and, in proportion as this undergoes the proper change, and is moved on by the muscular action of the stomach, that next in turn succeeds, to undergo the same change. Although the food is in the most digested state in the pyloric end, it appears, from several circumstances noticed by the author, that the change is chiefly effected in the great end of the stomach. "The food found in the pyloric end is comparatively dry, while that found in the great end, if digestion is much advanced, is mixed copiously with the fluids of the stomach; and there is a more evident difference in the state of the food, before it comes to this part, and when it is about to leave it, than in any other part of the stomach." This arrangement and progress of the food in the stomach, is also very clearly indicated by Dr. Copland, in his Observations on Human Rumination. The sensation experienced at the cardia previously to the expulsion of the bolus, the mechanism by which the undigested food is conveyed there, and the return of the food in the order in which it was taken, (that first swallowed being first disgorged,) are all explained by Dr. Copland in a way consonant with the observations of Dr. Philip: indeed, the observations in the paper alluded to and in the work before us serve to illustrate each other in a very interesting manner.

Keeping in view the foregoing account of the process of digestion in the rabbit, it will be interesting, Dr. Philip remarks, "to trace the effect produced on it by depriving the stomach of a great part of its nervous influence, by dividing the eighth pair of nerves."

"If the animal be allowed to live for a considerable time after the division of the nerves, the food remaining in the stomach, we have seen, if the animal has lately eaten, is always found undigested, and nearly in the same state in all parts of the stomach; a circumstance which I was at first greatly at a loss to explain. This effect was uniform; I never saw it otherwise. Yet we must conceive that, at the time the animal last eats, there is some food more or less digested in its stomach, and some gastric fluid to act on part of that just received into it. The foregoing statements explain the difficulty. The division of the eighth pair of nerves prevents the due formation of the gastric fluid; but, the animal still living, and the motions of the alimentary canal being independent of the nervous influence,* the usual motions of the stomach continue, and send onwards into the duodenum all the food which is digested, and consequently capable of applying to the stomach that stimulus which excites its natural motions.

"Thus, it is evident that the undigested food must at length come into contact with it. As soon as this happens, the usual secretions not being supplied to produce the proper change in the food, an unnatural

* Experimental Inquiry, chap. vi.

motion is excited: hence the efforts to vomit, which always ensue in about an hour, an hour and a half, or two hours, after the division of the nerves, marking the time when the stomach, having sent towards the pylorus its digested contents, begins to feel the effects of undigested food coming into contact with it. To these efforts to vomit we must ascribe the circumstance of food being generally found in the œsophagus when an animal dies from the division of the eighth pair of nerves; for food is found there, although it is not allowed to eat after the operation. If the animal be allowed to eat after the operation, the efforts to vomit almost immediately ensue; the food, as is evident from the way in which it enters the stomach, directly coming in contact with some part of the small curvature."

As we might be supposed to give our assent to the proposition, did we pass it over without any remarks, we just state, that we do not agree with the author that *the motions of the alimentary canal are independent of the nervous influence.* This is, we think, a disputable point: the known facts only serve to show that they may occur without any nervous communication with the brain and spinal marrow. Their dependance on the ganglionic nerves is still a probability.

Having thus given an exposition of the author's account of the process of digestion, and of the most important, in the present point of view, of the phenomena which result from diminished energy or paralysis of the stomach, we retrace our steps a little to notice some interesting observations on the digestion of the stomach after death by the gastric juice. Hunter's view of this subject has been disputed, to the present day, by several eminent pathologists; but Dr. Philip's observations are qualified to prove it in a very satisfactory manner to those who yet required convincing arguments. On opening the abdomen of rabbits which had been killed immediately after having eaten, and which were allowed to lie undisturbed for some time before the examination, he has found "the great end of the stomach soft, eaten through, sometimes altogether consumed, the food being only covered by the peritoneum, or lying quite bare for the space of an inch and a half in diameter; and part of the contiguous intestines, in the last case, also consumed, while the cabbage, which the animal had just taken, lay in the centre of the stomach unchanged, if we except the alteration which had taken place in the external parts of the mass it had formed, in consequence of imbibing gastric fluid from the half-digested food in contact with it.

"We sometimes found the great end of the stomach dissolved within an hour and a half after death. It was more frequently found so when the animal had lain dead for many hours. This effect does not always ensue, however long it has lain dead. It seems only to take place when there happens to be a greater than usual supply of gastric fluid; for we always observed it most apt to happen when the animal had eaten voraciously.

"Why it should take place without the food being digested, is evident from what has been said. Soon after death, the motions of the stomach, which are constantly carrying on towards the pylorus the most digested food, cease. Thus, the food which lies next to the surface of the stomach, becoming fully saturated with gastric fluid, neutralizes no more, and no new food being presented to it, it necessarily acts on the stomach itself, now deprived of life, and, on this account, as Mr. Hunter justly observes, equally subject to its action with other dead animal matter. It is remarkable that the gastric fluid of the rabbit, which in its natural state refuses animal food, should so completely digest its own stomach as not to leave a trace of the parts acted on. I never saw the stomach eaten through except in the large end; in other parts, its internal membrane is sometimes injured."

The author concurs with those who think it probable that the sense of hunger arises from the action of the gastric juice on the stomach; a supposition, he adds, which seems to be confirmed by the following experiment.

"A person in good health was prevailed upon to abstain from eating for more than twenty hours, and further to increase the appetite by more exercise than usual. At the end of this time he was very hungry; but, instead of eating, excited vomiting by drinking warm water and irritating the fauces. The water returned mixed only with a ropy fluid, such as the gastric fluid is described to be by Spallanzani, or as I have myself obtained from the stomach of a crow. After this operation, not only all desire to eat was removed, but a degree of disgust was excited by seeing others eat. He, however, was prevailed upon to take a little milk and bread, which in a very short time ran into the acetous fermentation, indicated by flatulence and acid eructations."

The author commences his disquisition on the remote *causes* of indigestion, by remarking that our attention must be as much directed to the sympathy of the alimentary canal with the other parts of the system, on this view of the subject, as it was in the consideration of its symptoms; for, he says, "as the whole system is essentially influenced by the affections of this canal, and some parts particularly so; in like manner it is influenced by the affections of the system in general, and particularly of those parts. The remote causes of indigestion, therefore, may be divided into those which act directly on the stomach and intestines, those which act on other parts, and those which affect the whole system." Another important general remark, which the author here adduces, is this, that—

"As the affections of parts which sympathize, whether primary or secondary, influence the state of each other, the sympathetic affections produced by disease become causes which support and aggravate it.

The debility of the skin, for example, occasioned by indigestion, so re-acts on the digestive organs as to increase the disease of the stomach. Similar observations apply to the affections of the liver, the brain, &c. produced by the diseased state of the stomach; and the disease is farther aggravated by the increase of general debility caused by these affections. It is thus that—the evil increasing, if I may be allowed the expression, in a geometrical ratio, and not by simple addition,—the whole powers of the system, in severe attacks of disease, often sink with a rapidity which at first view appears unaccountable. We shall find these facts strikingly illustrated in considering the causes and treatment of indigestion."

It had been previously stated that the sympathetic affections, amongst which gout was expressly mentioned, sometimes appear to relieve the stomach. Of the causes of this contrariety of effects we are wholly ignorant; but the circumstances, as above stated, are nevertheless apparently true. This is, too, one of the very few abstruse points in pathology on which no important light is reflected by the "General Indications which relate to the Laws of the Organic Life."

The functions of the stomach, the author infers, from what has been already stated, may be deranged in two ways: either by causes affecting its secreting power, so that the proper chemical change is no longer effected in the food; or by such as debilitate its muscular power, so that the food, though properly prepared as far as it is brought into contact with the proper parts of the stomach, is neither duly so brought, nor regularly propelled into the duodenum. The author believes that the motions of the muscular fibres are independent of the nervous influence, as we have remarked, though the powers and actions of the muscles may be altered by the medium of the nerves: and hence certain affections of the nerves of the stomach will derange the action of its muscular fibres. There are other causes of indigestion which, he thinks, act immediately on the muscular fibres: he says, citing his own words, "amongst the chief causes of indigestion, which act directly on the muscular fibres of the stomach, are narcotic and other offensive substances received into the stomach. I have found that, although opium applied to the external surface of the alimentary canal and heart, produces no sensible effect on their muscular power, applied to their internal surface it produces the same effect as when directly applied to the muscular fibres themselves,* immediately, unless the quantity be extremely minute, impairing their power, and instantly destroying it if the quantity be considerable.

"It may be questioned whether the opium pervades the fine internal membranes of these organs, and acts directly on their

---

* *Inquiry into the Laws of the Vital Functions.* Second edition.

muscular fibres, or affects these fibres through the nervous extremities distributed on this membrane. This is a question of little importance. I consider it as acting immediately on the muscular fibres, because its effect is the same as when directly applied to them, and different from what it is when it acts evidently on the nerves themselves; and we know that the bile is capable of transuding through the coats both of the gall-bladder and intestines."

Dr. Philip after this notices several other evident or probable causes of disorder of the action of the muscular fibres,—as distilled spirits, strong peppers, acid or putrid matters generated in the stomach itself, large quantities of warm or cold water, and distention.

"The most common cause of morbid distention of the stomach," Dr. Philip says, "is eating too fast; for, the appetite only subsiding in proportion as the food combines with and neutralizes the gastric fluid previously in the stomach, when we eat too fast time is not given for it to combine with that part of the food which is presented to it, till so much is taken that the whole gastric fluid which the stomach is capable of supplying during the digestive process, is not sufficient to effect the due alteration on it: whereas, when we eat slowly, so that a proper time is given for the combination to take place, the appetite abates before the stomach is overcharged; for, while digestion goes on, and the gastric fluid is only supplied in proportion as fresh food comes in contact with the coats of the stomach, it combines with the food as it is formed, and never excites the appetite."

Wine drank during dinner is apt, he thinks, to produce indigestion, by inducing the person to eat too much. Undue distension of the stomach, besides impeding the action of the muscular fibres, irritates the nerves, producing pain and sense of oppression, and, as a consequence of the nervous irritation, derangement of the secretory function: the proper chemical changes are, hence, not effected in the food, which therefore runs into fermentation or putrefaction; and, by swelling in this state, emitting gases, &c. the distention is increased by its own proper consequences. This is, probably, the most common order of the phenomena of indigestion; but other causes, the author remarks, "appear to make their impression wholly on the nervous system, only secondarily affecting the muscular fibres of the stomach;" several examples of which he adduces. The more remote impressions, acting on the surface of the body generally, or some particular organs, are then considered by the author; without, however, producing any important original remarks; so that we pass on to the section on the "*immediate causes of indigestion.*" The immediate causes of a disease he defines, "the states of body induced by the remote causes, and from which all the symptoms more or less directly arise." In endeavouring to trace the immediate causes of a disease, he adds, there are two objects which demand attention: the change produced in the seat of the disease by its remote causes, and the manner in which this change excites the symptoms. Without knowledge of the first of these, our principles of treatment must be vague; without that of the other, they cannot be adapted to the particular cases under treatment. The former of those points has already been considered: if the author's opinion of the express state of the stomach has not been already clearly manifested, it must be apparent in the following passage:—"In considering the remaining part of the subject, the manner in which debility of the muscular and nervous powers of the stomach produce the symptoms of indigestion, I shall, in the first place, consider those of the first, and afterwards those of the second, stage of this disease."

We have cited the author's own words, that we might avoid the possibility of mistating his meaning: if he has used the term *debility* in this sentence in a more general way than he intends, it is not our fault if his views of this part of the subject should appear to be too confined, seeing that we have found no other passage in which he directly modifies this general assertion. The difference between the author and us may, however, consist merely in what relates to the signification of a word: where the functions of an organ are so deranged as not to produce their ordinary effects, the organ may, as far as regards those functions, be said to be in a state of *debility*, even though certain other actions of it are exalted. Medical men, in general, are, however, accustomed to apply the term debility to a state of an organ very different from that in which there is inflammation; which is, undoubtedly, often the case with indigestion. In this view, however, it should be borne in mind, that Dr. Philip considers inflammation as a state of relative debility; or, more precisely, of relative debility of the vascular part of the structure in which it is seated.

With regard to the symptoms which are confined to the stomach itself, the author observes, that little need be added to what he has already said; "as the food can only be regularly propelled into the duodenum by the due action of the muscular power of the stomach, it is evident that, if this fails, oppression and distention must ensue; and, if the due secretion of the gastric fluid depends on the healthy state of the nervous influence of the stomach, its properties must be affected by any cause disordering this influence, and the food, consequently, will not be duly changed by it."

The mode in which the symptoms in remote parts are produced, is not so evident as that of the local affections. The author commences his disquisition on this point with some considerations on the laws of what is termed sympathy. On discussing the question, whether the sympathy which exists between different parts depends "on the connexions in the course of their nerves," or is produced through the medium of impressions on a nervous centre, as the brain or spinal marrow, he admits that "it is possible, however, that some collateral facts may prove that the former is the just explanation;" and yet he himself expresses a positive belief that the latter is the true one: he says,

"These and various similar facts, as far as I can judge, leave no room to doubt that nerves sympathize only from their connexion in their common source, and that the numerous connexions we observe in their course are only useful in the same way with the ganglions and plexuses, which may be proved by direct experiment, to enable the influence, descending from that source, to pass from one nerve to another, so that one may partake of that which is conveyed by many; a power which, it may also be shown, is necessary to the continuance of life.* That the phenomena of sympathy depend on changes in the source of nervous influence, might be proved by the fact alone that sympathetic feelings still continue to be referred to a limb which is lost."

It is the following *queries* that are referred to at the commencement of the foregoing paragraph, under the appellation of *facts.*

"Is it found that parts never sympathize unless their nerves are connected in their progress? Do parts whose nerves are most connected, most sympathize? A crowd of facts reply to these questions. What connexion of nerves exists between a vital organ and the skin which covers it,—between the liver and the ligaments of the shoulder,—between the viscera of the abdomen and its parietes, the stomach and the cartilages of the ribs, &c.? Why does inflammation of the pleura of the ribs spread as readily to that of the lungs, which is only in contact with it, as to that in continuation with it, which is supplied from the same branches both of nerves and vessels? Why is inflammation of the parietes of the abdomen, or of any part of the bowels, in like manner, as readily communicated to the part in contact with it, however little their nervous communications, as to that with whose nerves the nerves of the affected part most freely communicate?"

Before these arguments, if they may be so termed, can have any force, it must be proved that inflammation of the pleura, and the other parts subsequently noticed, does spread as readily to merely contiguous, as it does to continuous, parts: and, in expressing our dissent to the proposition, we believe that we

* *Experimental Inquiry,* part ii. chap. 4, 5, and 7; sect. ii. and chap. 9.

agree with the generality of physiologists. BICHAT points out (*Anatomie générale*, Considerations générale, § vii.) instances of extension of disease from one tissue to another which is only contiguous, as casual exceptions to general laws.

It is our duty to give a view of the author's opinions on the chief points relative to the subject; but it is very irksome to be constantly citing conclusions founded on assumed *facts*, which are merely Dr. Philip's *inferences:* because, where the correctness of these inferences is disputable, the conclusions must, of course, be disputable also. In continuation from the penultimate paragraph, transcribed from the work before us, the author says,

"If we compare the foregoing facts* with the result of experiments which prove that the vital organs are supplied with nervous influence from every part of the brain and spinal marrow, while the organs of sense derive theirs only from particular parts of them, it seems a necessary consequence that the one set of organs cannot be injured without influencing every part of the system; while, in injuries of the other, only those parts of the general source of nervous influence from which their own nerves arise being affected, the evil extends no farther."

There is not, we think, any thing like a proof of the proposition that "the vital organs are supplied with nervous influence from every part of the brain and spinal marrow." But we shall leave our readers to consider the arguments for it, referred to by the author, in the "Experimental Inquiry." We have neither space nor inclination for the discussion of these points. Dr. Philip's mode of reasoning and his manner of discussing a subject, in physiology, are such as will prevent our considering his disquisitions of this sort in a serious manner; and we should not have occupied ourselves so much with the point just noticed, were it not that the author founds on his propositions an inference of considerable importance in regard to the express subject of this work. He says,

"It appears from what has been said, that juxta-position is one of the most powerful causes of parts partaking of the affections of each other. We see textures of the most dissimilar nature, receiving their nerves and vessels from the most distant sources, immediately partaking of each other's affections if they lie together. Inflammation of the skin of the chest often spreads to the intercostal muscles, to the pleura of the ribs, to that of the lungs, to the lungs themselves. The same observations apply to the other cavities, for the interposition of bone itself does not always prevent this progress. This well-known fact is of great consequence in tracing the causes and explaining the phenomena of diseases. We shall find it so in the disease before us."

* We have transcribed all that Dr. Philip has adduced as facts bearing on this point.

This last remark of the author seems to be made in regard to the disease of that part of the liver which is in contact with the pylorus, that so frequently, and especially, as the author thinks, occurs in indigestion. After having noticed the influence of the morbid state of the nerves of the stomach, and, as a consequence, of the nerves more generally, and the want of due nutrition, in developing remote affections; and repeating the remark, that sympathetic nervous irritation may, and generally does, when it is of long continuance, terminate in inflammation; without, however, adducing any thing that is remarkable for novelty, he says,

"It is evident that, of the different parts of the stomach, the pylorus is the one most exposed to the causes of irritation. Other parts experience the irritating effects of different portions of its morbid contents, but the pylorus is necessarily exposed to those of all. All must pass by this orifice. When therefore we see that, after indigestion has continued for some time, a certain part of the region of the stomach becomes tender on pressure, we cannot help turning our attention to this part. Now, in the natural situation of the viscera, exactly in the tender part of the epigastrium, the pylorus lies, with the thin edge of the liver upon and in contact with it; as I have ascertained with the kind assistance of Mr. Brooks, whose anatomical skill is so generally acknowledged.

"When all these circumstances are considered, can we doubt that it is the irritated pylorus assuming a low degree of inflammatory action which occasions the tenderness on pressure above described; and, when we consider what has just been said of the influence of juxtaposition in the spreading of this disease, can we doubt that, when we find the tenderness, with some degree of fullness, gradually extending downwards along the soft parts on the edge of the cartilages of the right side of the epigastrium, as we find it to do in the progress of indigestion, and at length ending in evident enlargement and tenderness of the liver, that the affection of the pylorus is communicated to the thin edge of this organ, with which it is in contact, thence by degrees extending to its other parts? Thus it is that, of all the neighbouring parts, no other so frequently partakes of this affection of the stomach. It is, doubtless, in the same way, that the habit of drinking spirits, which must apply so great a degree of irritation to the pylorus, seldom fails to produce affections of the liver."

The author takes care to remark, that fullness and tenderness in the right hypochondrium, in indigestion, does not always indicate disease of the liver; it sometimes originates from undue distention of the duodenum. On considering the means of distinguishing the two affections, he says,

"It has been stated, in enumerating the symptoms, that the fullness and tenderness of the hypochondrium in the early stages of Indigestion are often temporary. I have repeatedly seen them, even when they were considerable, disappear after the operation of a brisk cathartic. They must then arise either from a distended and oppressed state of the duodenum, or temporary distention of the various vessels of the liver. To which of these causes we should ascribe them, the accompanying symptoms will generally enable us to determine. The feeling given to the hand by the distended duodenum is different from that produced by the gorged liver, and in the former case the chief fulness is generally lower down, and does not seem to proceed so immediately from under the edge of the thorax as general fulness of the liver does; so that, even where, from more permanent debility of the duodenum, its morbid distention is a more constant symptom, the two cases may generally be distinguished. The more temporary nature of both these affections in the generality of cases, with the history and general symptoms of the case, for the most part readily enable us to distinguish them from fulness and tenderness of the same part arising from organic disease of the liver, which comes on slowly and uniformly; and the commencement of which, when it arises from Indigestion, may generally be traced to the epigastrium.

The author, in this place, takes occasion to adduce his notions of the nature of Inflammation, with not less confidence of their truth than that which he entertains of his other hypotheses, already noticed, and without seeming to think it possible that he can have drawn false inferences from the results of *experiments.* He says,

"It appears from many experiments, detailed in the Introduction to my *Treatise on Symptomatic Fevers,* and which have been carefully repeated by Dr. Hastings* with the same results, that all the causes of inflammation act by debilitating the capillary vessels of the part, and thus allowing them to be distended by the *vis à tergo,* from which, as appears from what is said in the above Treatise and last part of the *Inquiry into the Laws of the Vital Functions,* all the phénomena of inflammation necessarily arise.

"It is this debility of the finer vessels of the pylorus, therefore, produced by long-continued irritation of its nerves, from which arise, in the second stage of Indigestion, the hard pulse and other symptoms of feverishness above enumerated; and by which, we shall find, the treatment of this stage must be essentially influenced."

This doctrine is followed by an hypothesis of the difference between acute and chronic inflammation.

"It is a point of great importance in the progress of disease, that febrile disease begins with inflammatory action, that is, debility of the finer, and consequent increased action of the larger, vessels, and at length necessarily produces debility of the nerves; while nervous disease begins with the latter, and as certainly ends in debility of the finer vessels. It seems to be this which renders the chronic inflammation of organs so much more difficult of cure, and apt to run to derangement of structure, than the acute form of the disease. In the

* Dr. Hastings's *Treatise on Bronchitis.*

former, the functions of the part are more generally injured. Its nervous power is debilitated before its vessels begin to be distended. Besides, the debility of the nerves, and that of the extreme vessels, tend to increase each other; and, unless the chain of diseased action can be broken by means which enable the vessels to recover their healthy diameter, it goes on more or less quickly, sometimes, if the symptoms are mild, very slowly, till the structure necessary to the function of the organ is wholly destroyed.

The next and last of the effects, or general symptoms, of indigestion, noticed by the author, are those affecting the fluids of the body. He says,

In proportion as the flow of nutriment into the blood is lessened, the chyle itself, probably, more or less vitiated, and as the different secretions fail, the circulating fluids must be subject to various changes. What these are, the present state of our knowledge makes it impossible to ascertain.

In those, who have long laboured under Indigestion in its more severe forms, the blood is sensibly altered in some of its properties. The proportion both of red globules and lymph, is less than in health. This state of the blood must necessarily affect that of the fluids supplied to secreting surfaces, and from this cause also, those of the digestive organs must further deviate from the healthy state.

We now arrive at the part of the work which relates to the treatment of Indigestion; but, as we have not at present space left for all we wish to notice on this subject, we shall defer entering on it till the next Number. Though we acknowledge that the author's history of the symptoms of Indigestion is remarkably perspicuous and apparently satisfactory, as far as it extends, and are disposed to attribute a high degree of importance to what is especially his own, the account of the phenomena, at least of the palpable phenomena, of digestion in the healthy state, and the two chief ways in which the stomach may fail in fulfilling its office; we are disposed to consider what relates to the treatment as by no means an inferior part of the work, in regard to its merit. We differ from the author on several points of physiology, and think his general mode of reasoning—that of proceeding on his own inferences as if they were indisputable facts—and his ambiguous statements in some instances, are better qualified to produce interminable polemical discussions, than to improve the science of physiology; but we are not, therefore, we hope, the less sensible of the value of the very great extent of the original and interesting observations, and the precise and excellent therapeutic precepts which characterize his dissertation on the treatment of the diseases which are the subject of his work. His dissertation evidently presents the results of very acute and careful observance, and of well-cultivated and long experience in the management of those diseases, and is qualified, we believe, to effect a very considerable degree of improvement in their cure.

---

*A Treatise on Stricture of the Urethra; containing an Account of improved Methods of Treatment: with an Appendix, noticing the Application of a new Instrument to the Treatment of enlarged Prostate Gland, Gleet, Fistula, and other Diseases of the Urethra, Œsophagus, and Rectum.* 8vo. pp. 183; with four Plates.

*Cases illustrative of the Treatment of Obstructions in the Urethra, &c. by the new Instrument, the Dilator, with further Directions to facilitate its general Adoption; also, a Case of Extraction of Stone from the Male Bladder, without cutting it, by the Dilator; with an Account of Improvements of the Method of dissolving Stone by Injection, and of the common Operations of Lithotomy.* By James Arnott, Member of the Royal College of Surgeons in London. 8vo. pp. 119; with one Plate. Burgess and Hill, and Longman and Co. London. 1819 and 1821.

If we have delayed the review of these works until we have obtained some practical experience of the efficacy of the measures they advocate, we are confident the public, as well as the author, will, on that account, attach a higher value to our opinions, and will derive a greater satisfaction from the approbation both justice and our duty require us to bestow upon them. These measures, in truth, present such improvements in the cure of strictures of the urethra and other membranous canals, by means of Dr. N. Arnott's instrument, the dilator, as leave little doubts in our minds of their forming a new era in the treatment of these complaints, and of their conferring such benefit on the public, and such advantages on the profession, as will ensure their general adoption. Nor can it be denied that improvements were wanting in this branch of surgical practice, when we consider the tedious and imperfect treatment by the bougie, and the occasionally dangerous and fatal effects of the application of caustic. With these convictions, we shall proceed to give a short analysis of those portions of each volume which contain sentiments peculiar to the author, or inventions assignable to him or his brother, and which will so entirely occupy the space we can allot to them, as to almost prevent us from glancing at "the systematic account of stricture."

Although we agree with Mr. A. in thinking his treatise complete as well as concise, and perfectly coincide with him in the opinion that membranous canals are endued both with elasticity and contractility, still we must pass over his five chapters, of the Anatomy of the Urethra, of the Nature, of the Causes, of the Symptoms, and of the Diagnosis, of Stricture, as not offering any particular novelty, or any thing which the profession is not acquainted with. In chap. vi., on the Prognosis of Stricture, we had expected to be informed what length of time, and what number of applications, experience had proved to be necessary to the cure of stricture by the new method; and in the diminution of both we had no doubt but that the superiority of the dilator to the other instruments in use would have appeared very manifest. This deficiency has been partly supplied by the seven cases detailed in the second pamphlet, all of which were cured by a few applications within a month; and, in our own practice, we have succeeded in permanently dilating and curing thread strictures by two applications of the dilator, and by passing a No. 13 bougie, or sound, a few times afterwards. We need not mention, that from twelve weeks to twelve months have been usually required for the performance of cures by the bougie.

In ch. vii. sect. 1, the author points out the various means of cure that have been employed by the profession; and then states, that the principle on which the dilator acts is that of dilatation, similar to the bougie, but its action, nevertheless, differs from it, and obviates all the imperfections and disadvantages of the bougie, as will be explained in the sequel.

"As surgeons," says the author, "have believed that, where dilatation could be properly made, no stricture would resist it, the great desideratum was an instrument which could be easily passed through a stricture, and any part of which could be then increased in diameter, to any size and with any force; and be again reducible to its primitive small bulk, when the operator should wish to extract it."—"Dr. Arnott's dilator completely supplies this desideratum."—"It consists of a strong, air-tight, membranous tube, as of oiled silk lined with thin gut, about an inch and a half in length, which is introduced into the stricture in its empty or collapsed state, and is then filled to the necessary degree of pressure, with air or water, from a syringe without; and is again emptied before being withdrawn. The dilator, while opening the stricture, remains precisely in the same position within it; so that, however strongly its action may be required or exerted, even when an old stricture is opened by it at one application, it cannot possibly, like bougies, either pierce the urethra or tear it. As it is introduced in its shrunk or collapsed state, no painful or injurious friction is then occasioned; and, from its being changeable in dimensions, it will enter an urethra with the narrowest orifice, and still dilate a contraction in any part to the natural size, or beyond that, if necessary, without stretching, like the bougie, the whole canal anterior to it. For the same reason, the dilator acts equally on the whole of a long stricture or on several strictures at once. One end of the tube of silk, lined with catgut, is tied upon the extremity of a directing wire, which runs through and projects beyond a canula, and the other upon the extremity of the canula. The wire serves to conduct the dilating tube, in its collapsed state, into the stricture; and, by the canula, the distending fluid, air, or water, is injected from a brass forcing syringe into the silk tube or dilator, and is there retained by a stop-cock screwed into the outer end of the canula. The end, or small pipe, of the syringe is, of course, inserted into the canula."

In both works, the author has given accurate plates of the apparatus, with a description.

It is sometimes of considerable importance to ascertain, "not only the length of strictures, but their number and relative situations;" circumstances to which little attention has been paid, from the want of adequate means of discovery, and from our inability of removing more than one stricture at a time by the old methods of treatment. The author remedies these defects by a dilator-sound, whose "canula should be stiff and very narrow, and the bag very short, nearly of the natural diameter of the urethra, with its extremities as flat as possible.

"This is introduced to the first stricture, and the distance from the orifice of the urethra observed: letting out the air, it is then passed through the narrowing, again distended and retracted till the posterior surface of the same stricture opposes it; the distance of this from the end of the canal is then marked, and the space between this mark and the former shows the extent of the urethra occupied by the stricture. The instrument is now passed to a third stricture; the same process repeated, if deemed necessary; and so on, till the whole length of the urethra is examined."

Notwithstanding the advantages of the dilator, in all ordinary cases, over every other mode of practice, the success of its use is neither certain nor rapid when a stricture is met with whose parietes are of an almost cartilaginous hardness, without elasticity or contractility. For such instances, the application of caustic still seems necessary; and Mr. A. has devised a new method of applying it to the whole length of the stricture, instead of to the anterior part only. This is effected by piercing with a wire, through its axis, a bit of caustic, of a diameter rather less than the stricture, which is conducted exactly within the stricture through a full-sized canula passed down to it.—P. 142.

At p. 155, a method is described of cutting out a circular portion of stricture, (we suppose, of the short or thread kind,) by one push and turn of a circular knife, like the trephine.

"Before cutting, the urethra before and behind the stricture is fully distended,—the outer portion by a large canula introduced down to the stricture, and the inner by a dilator which has been passed through it, and drawn close against its posterior surface, so that the stricture itself remains compressed between these two, and projecting inwards. The knife, then, like a second canula issuing from the first, makes its way through to the dilator, and detaches the whole of the stricture, which comes away in its tube."

The dilator is then drawn forward upon the newly-cut surface, to stop the hemorrhage. This practice the author thinks applicable "where the stricture has a peculiar tendency to return; where it is important to obtain immediate relief, to obviate bursting of the canal; or where the narrowness of the stricture, its spasmodic nature, or the existence of a false passage, renders the attempt to cure by dilatation abortive." In the latter instances, we apprehend there must be great difficulty in passing the dilator beyond the stricture.

In an Appendix, the author enumerates the principal surgical diseases in which the dilator seems applicable: these are, strictures of the œsophagus and rectum, of the latter of which a successful case is recorded at p. 72, vol. ii.; urinary calculus; fistulas in perinæo and ano; hemorrhage from the nose; menorrhagia; inward piles; prolapsus uteri and ani.

In the second pamphlet, the author points out, in an Introduction, what he terms the four radical defects of the mode of dilatation by the bougie, catheter, &c. from which the dilator is found to be free. The two principal of these defects are, that the bougie often pierces the urethra before or behind the stricture, causing hemorrhage, false passage, and urinary abscess, and "that such an unchangeable instrument cannot act equally on the whole of a long stricture, or on several co-existent, at once."

The second section contains an explanation of the plates of the dilator, and a description of the manner of constructing the instrument; and the third section a description of the manner of applying the dilator.

"One difficulty," says the author, "which surgeons may experience in commencing the practice of the dilator, is to know when they have got the distensible tube exactly in the stricture. By careful previous measurement, the object may in general be secured; but, where there is doubt, the end of the conducting tube may be enlarged, so as to form a kind of button; and, when this is obstructed in passing on by the stricture, the silk tube must be within it. Or again, a short distensible tube, without such a button at its outer extremity, may be passed beyond the stricture, then filled and retracted, till this impede it: allowing then the fluid to escape, and withdrawing the instrument half an inch farther, it will be within the stricture. After thus placing the distensible tube accurately, the syringe has now to be applied, and the patient may keep the tube steadily in the proper position, by resting the wrist of the hand employed upon his groin: he must retain it so, if the pressure from the injected fluid is intended to continue for some time."

"Continued gradual pressure is best made by injecting air, the elasticity of which continues the dilatation as the stricture gives way, and yields to any momentary violent spasm of the parts; and more is afterwards injected, or part allowed to escape by the cock, according to the patient's sensations. If the dilatation is intended to be sudden and momentary, then the injection of water will, on several accounts, be preferable to air."

Gradual continued dilatation seems the best adapted to the long species of strictures, and the sudden, full, and complete dilatation, to the short or thread strictures; and, indeed, in a large proportion of the varieties of stricture, momentary and considerable distension by the dilator is the best and most effectual method of treatment.

It may answer, says the author, in some cases, to dilate the stricture at once from a very narrow state to the full size of the urethra; but, when there is great iritability of the urethra, or the testes or perineum are disposed to inflammation, it is the better plan to employ a gradation in the sizes of the dilator, and increase their dimensions with some caution.

The introduction of the dilator produces ardor urinæ, a discharge from the urethra, and other effects, similar to those occasioned by the use of the bougie; and the instrument should not be re-applied until the irritation excited by it has nearly or quite subsided. When the use of the dilator is followed by a slight hemorrhage, it may be regarded as an advantageous circumstance; for it seems to indicate that the membrane forming the stricture has been ruptured.

After the stricture is dilated to its fullest extent by a dilator of about one-third of an inch diameter, and this has been *quickly* effected, "it will probably, in most cases, remain permanently distended." Nevertheless, the author advises the use of the dilator to be gradually relinquished, in imitation of the practice where the bougie is employed: we may however observe, from our own experience, that the use of the dilator, or bougie, as a substitute, need not be continued but for a short period, when compared to the length of time it is ordinarily and necessarily persevered in after the cure by bougie-dilatation; and the intervals between the periods of introduction may be made much longer.

After these general rules and observations on the dilator have been made, the author details eight cases successfully treated by it; and then offers some observations "on Stone in the Bladder."

There is not, perhaps, a disease that has more powerfully excited the attention of surgeons and chemists, than stone in the bladder. Surgeons have not only sought with earnestness opportunities of performing lithotomy, and have been peculiarly proud of their success in it; but physicians and chemists, encouraged even by legislators, have assiduously engaged themselves in discovering solvents for the stone, and the means of preventing its formation and growth: yet the former have not arrived at any uniformity of practice; they have not decided whether the high or the low operation is to be preferred, nor have they agreed to what kind of instrument for cutting into the bladder they should assign the superiority: while the latter have not devised the means of dissolving calculi in the bladder, although Dr. Marcet has ascertained that the bladder will retain, for an hour, without inconvenience, chemical solvents sufficiently strong to dissolve an earthy calculus. As urinary calculi are soluble out of the body, precisely the same results should follow the constant application of these solvents to stone in the bladder; and, for this purpose, Dr. N. Arnot has contrived—

"A double catheter, which may be made of metal or of elastic-gum. When of metal, it is formed by running a partition along a common catheter, so as to divide it into two channels, which open near its point, by distinct holes of the usual size. By one of these channels, liquid may be passing into the bladder, while it is again escaping, mixed with the urine, by the other. When of elastic gum, it is formed by inserting a small catheter into a larger one, and using the first for the injection of the fluid, while the latter allows it again to run off. Separate flexible tubes must be attached to the outer extremities of the divisions, as prolongations of these: one, to connect the catheter with the reservoir from which the fluid is to enter by it; the other, to carry off the waste fluid and urine to a fit receptacle."

In his remarks on lithotomy, Mr. A. observes, that the subsequent escape of urine by the wound, both in the lateral and, more particularly, in the high operation, is a great cause of fatal terminations.

"Now," says he, "a catheter constructed so as to act as a syphon, introduced by the urethra, will carry off every drop of urine by the natural outlet, as soon as it descends from the kidneys; and, instead of leaving a tendency in the urine to spread in the wound and adjoining parts, the action of the instrument may be made so strong as even to draw any secretion from the wound that may take place in it."—"The syphon-catheter requires to be longer than a common one, and should have several openings in the point: an inch of its external extremity must be turned sharply up, that it may always remain full of urine; for, should the air get into it instead of liquid, it ceases to be a syphon, and acts only as a common catheter. It should be filled with water before it is introduced, or immediately after."

The urethra should be accustomed to the presence of the catheter before the operation, and the syphon-catheter should be retained by something passed through it, which, on projecting from its inner open extremity, will expand in the manner of the urethra spring-forceps, and act as "a button to keep it in its place. It might also be a useful precaution to tie a bit of gut upon the outer extremity of the syphon, which would constitute a valve, allowing the escape of the urine, but, by its collapse, preventing the access of air."—" It is obvious, also, the use of the syphon-catheter will facilitate the healing, by allowing the parts to be perfectly at rest."—P. 24.

In the high operation, the author thinks, there is another desideratum required, besides the means of preventing the escape of urine by the wound ; and this is a contrivance to enable the surgeon " to cut into the bladder readily, without the risk of wounding the peritoneum." Mr. A. proposes to effect this " by a staff, or strong catheter of the common form, made to contain in its curved part a sliding piece, that may be pushed out after its introduction, like the joint of a telescope; the curve would thus be lengthened to nearly a semicircle, and, of course, the point might easily be felt above the pubis." This would necessarily present to the surgeon the part of the bladder to be operated upon.

In conclusion, Mr. A. details a case, where the entrance to the bladder was dilated by an air-dilator, passed through an opening made, by Mr. Cooper, into the urethra from the perineum. A dilator of half an inch diameter was first introduced; and, after twenty hours, one of three-quarters of an inch diameter was substituted, and retained in the passage till the forceps was introduced, and a flat oval stone was extracted, " of the size of a walnut."

Our limits compel us to pass over many excellent observations and some ingenious suggestions and inventions,—such as the description of the means of procuring a sufficient quantity of any calculus to enable us to determine its chemical composition, and the proper solvent to be injected by the double catheter ; the scale for measuring the size of bougies, &c. We, however, do not feel uneasy at our omissions, as we are persuaded every surgeon who is desirous that his knowledge shall be co-extensive with modern improvements will read the original works, in which he will find much to exercise his mind and recompense him for his perusal ; and from them he may learn how much practical advantage a surgeon may derive from the study of, and familiar acquaintance with, the philosophy of matter, and a judicious application of such knowledge to the art of surgery, in imitation of the example before us.

*Practical Observations on the Use of the Cubebs, or Java Pepper, in the Cure of Gonorrhœa. With Cases.* By HENRY JEFFREYS, Esq. senior Surgeon of the St. George's and St. James's General Dispensary; assistant Surgeon to the Lock Hospital; and formerly a Surgeon in the 3d Regiment of Guards. 8vo. pp. 67. Callow, London. 1821.

THE author has chosen to make a *book* of his "observations" and "cases," with the assistance of a common-place account of the more general points in the history of gonorrhœa; a botanical description of the cubebs, taken from well-known authors; and Vauquelin's analysis of the remedy, that has appeared in almost all the Medical Journals. The book is published, he says, "*in the absence of more elaborate information on the subject;*" and he also observes, in the Preface,—alluding to the remedy,—that "no detailed account of its agency has yet been given to the world;" by which it would appear that we have much mistaken the character of the papers on this subject in the Edinburgh Medical and Surgical Journal: a mistake which has led us to decline having our Journal occupied more than it has been by what we regard as details, unless it should be to relate something really novel respecting the effects of the medicine.

The following passages comprise the author's inferences from his observations:

"Taken into the stomach, this medicine does not appear to produce any very particular or predominant action on the system. Like other spices, it is warm and stimulating. In some constitutions it proves mildly aperient, probably by giving tone and vigour to the bowels, in the same way as the celebrated Ward's Paste: in others it has a contrary effect, and castor-oil, or some other gentle laxative, is required to be taken occasionally during its exhibition, in order to keep the bowels regular. In some persons, to whom I have administered the cubebs, it has produced a considerable degree of head-ach and nausea; and in one instance (Case xx.) the patient was so annoyed by these symptoms as positively to refuse going on with the remedy. When given in large doses,—as a drachm and a half or two drachms of the powder* four times in the day,—it appears to increase the secretion of urine, which assumes a deeper tinge of colour than usual, is voided somewhat more frequently and in larger quantity at a time, and acquires a peculiar and slightly-aromatic odour, which is not unpleasant.

"Of the mode in which this medicine operates in the cure of gonorrhœa, it is not easy to give a satisfactory explanation. Its agency appears to me to resemble, in great measure, that of copaiva; but, whether it is conveyed to the seat of the disease directly, by means of

* "The powder of cubebs varies in strength according to the quality of the berries, and their freedom from *stalks* and other impurities; but I have not detected any important adulteration in the specimens which I have examined."

the urine, or indirectly through the general circulation, or in both ways, I will not take upon myself to decide. It possesses, however, what may justly be called a specific power in most constitutions, especially when administered in the early and acute form of the disease. It moderates the inflammatory and most painful symptoms, and suppresses the quantity of the discharge in a shorter time, and with more certainty, than any other remedy with which I am acquainted. I have reason to believe that its exhibition is rarely, perhaps never, followed by any of those consequences which so often take place under the more ordinary modes of treatment, when the discharge is suddenly checked, —such as irritation in the bladder, strangury, swelled testicle, &c. It has not, at least, occurred to me to witness any of these accidents in my experience of the remedy; and, as far as I have been enabled to observe, it is in the more inflammatory forms of the disease in which its efficacy is most certainly displayed.

"The good effects of the cubebs pepper, in cases where it is of service, commonly begin to manifest themselves within forty-eight hours after the exhibition of the first dose: and I think that, in the generality of cases, unless some material relief is obtained in the course of five or six days, its continued administration will rarely be attended with advantage. The period in which it has effected a cure, in those cases wherein it has succeeded under my management, has varied from two or three days to a fortnight or more. In many instances it has proved to be of considerable efficacy where other remedies had been tried in vain, and where the disease was of some standing. Out of the first twenty-one cases in which I prescribed it, taken indiscriminately as they occurred, fourteen were cured, four relieved, and in three it failed. Of those in which it appeared to afford only a partial relief, the want of complete success may fairly be attributed to some irregularity or inattention in the patient's mode of living, or to a carelessness in taking the medicine. Nevertheless, one important advantage has appeared to me to be derived in these cases from the administration of the cubebs,—which is, that the symptoms afterwards yielded with greater facility to copaiva than under ordinary circumstances. In the instances wherein it failed entirely, the disappointment may have depended on some peculiarity of constitution; but, from whatever cause it may have arisen, the proportion of failures has been far short of that which occurs under the more common plan of treatment.

"There appears to be no good reason why the cubebs should not be combined with any of the other medicines commonly prescribed for the cure of gonorrhœa: in a few cases I have given it in conjunction with nitre, with the view of profiting by its diuretic properties; but at present I am not prepared to say that any material advantage was derived from the combination. When the more urgent symptoms had been allayed, it has appeared to me that the cure has been accelerated by the use of a mildly-astringent injection.

"One of the most common, and perhaps the most intractable, of the sequelæ of gonorrhœa, under ordinary circumstances, is gleet; but I think this will be found to be a less frequent consequence of the disease when it has yielded to a course of cubebs."

The author gives details of twenty-seven cases, " taken," he says, " without any particular selection, almost in the order in which they occurred to my observation." Of those twenty-seven cases, eighteen were cured, (and six relieved,) in periods varying from two to eighteen days: the medium period of the whole eighteen cases,—not, of course, calculating on two of them which are said to have been cured in " a few days" and " a short time,"—was eight days. In the case cured in two days, and in two others, the patient also used injections. In one of the cases, cured in fourteen days, the disease had existed " for several months;" but it was merely, as the account is given by the author, " a gleety discharge," for which injections had been used, and which had " very considerably increased in quantity," after the patient " had travelled from Cambridge to town on the outside of a carriage." In another case, cured in eight days, the patient had " had symptoms of gonorrhœa for three months, and at the time of his admission there was a thin gleety discharge from the urethra, with some degree of ardor urinæ and chordèe. He had been under treatment, and had taken a good number of pills, without much benefit."

Of the three cases of failure of the medicine, in one " the disease was of two months' standing, and had degenerated into a gleet;" over which affection, the author says, according to his present experience, it exercises much more limited powers than over a recent gonorrhœa. In another of the three cases, " the disease was simply an increased secretion of the natural mucous discharge of the urethra, in consequence of irritation from the long-continued excitement of lascivious ideas. In this case the medicine cannot be said to have had a fair trial, as it was left off before there could have been time for the full development of its powers." In the third, the author says, " the medicine would have had a better chance of success if the patient had lived quietly, and confined himself to the house."

As far as the author has hitherto been able to ascertain, there is no symptom which contra-indicates the use of cubebs. " I have not scrupled," he says, " to prescribe it in all the stages and under all the circumstances of the complaint, and as yet have had no reason to repent of so doing."

We have given an account of the whole of the interesting information presented in this book, excepting that, if there be any,—considering what had already been written on the subject,—comprised in the details of the cases. Every man has, of course, a right to publish any thing he may wish to promulgate in whatever way he likes best; but, when the information is so small, and that little so devoid of remarkable novelty as that contained in this book, we think it would be better, and more conformable with the custom of the more esteemed part of the profession, to make it the subject of a paper for a Medical Society or a Medical Journal, than—with the aid of large types and other means, which printers, for their own profit, know so well how to make the most of,—to expand it into a book which shrinks almost to nothing when we would grasp what it contains. There are, it is true, uses in a book which are not possessed by a paper published in the way above mentioned; but these are uses which do not appertain to the most assured mode of conveying to the medical profession, as extensively as possible, the sort of information comprised in that of Mr. Jeffreys.

---

